# Community Assembly and Co-occurrence Patterns Underlying the Core and Satellite Bacterial Sub-communities in the Tibetan Lakes

**DOI:** 10.3389/fmicb.2021.695465

**Published:** 2021-09-17

**Authors:** Qi Yan, Jianming Deng, Feng Wang, Yongqin Liu, Keshao Liu

**Affiliations:** ^1^School of Life Sciences, Lanzhou University, Lanzhou, China; ^2^Center for the Pan-Third Pole Environment, Lanzhou University, Lanzhou, China; ^3^State Key Laboratory of Grassland and Agro-Ecosystems, School of Life Sciences, Lanzhou University, Lanzhou, China; ^4^State Key Laboratory of Tibetan Plateau Earth System, Resources and Environment (TPESRE), Institute of Tibetan Plateau Research, Chinese Academy of Sciences, Beijing, China; ^5^College of Resources and Environment, University of Chinese Academy of Sciences, Beijing, China

**Keywords:** core and satellite sub-communities, biogeographic patterns, community assembly, co-occurrence patterns, Tibetan lakes

## Abstract

Microbial communities normally comprise a few core species and large numbers of satellite species. These two sub-communities have different ecological and functional roles in natural environments, but knowledge on the assembly processes and co-occurrence patterns of the core and satellite species in Tibetan lakes is still sparse. Here, we investigated the ecological processes and co-occurrence relationships of the core and satellite bacterial sub-communities in the Tibetan lakes via 454 sequencing of 16S rRNA gene. Our studies indicated that the core and satellite bacterial sub-communities have similar dominant phyla (Proteobacteria, Bacteroidetes, and Actinobacteria). But the core sub-communities were less diverse and exhibited a stronger distance-decay relationship than the satellite sub-communities. In addition, topological properties of nodes in the network demonstrated that the core sub-communities had more complex and stable co-occurrence associations and were primarily driven by stochastic processes (58.19%). By contrast, the satellite sub-communities were mainly governed by deterministic processes (62.17%). Overall, this study demonstrated the differences in the core and satellite sub-community assembly and network stability, suggesting the importance of considering species traits to understand the biogeographic distribution of bacterial communities in high-altitude lakes.

## Introduction

In natural ecosystems, bacteria within a metacommunity could be partitioned into different ecological assemblages, such as abundant or rare sub-communities and core or satellite sub-communities in light of potential importance for the community function (Unterseher et al., 2011; Jeanbille et al., 2016; Lindh et al., 2017). Defining OTUs as abundant and rare taxa are often conducted on the relative abundance of each taxa (Campbell et al., 2011; Alonso-Sáez et al., 2015), while the division of the core and satellite taxa is based on occurrence in addition to abundance (Magurran and Henderson, 2003; Hu et al., 2017b). The latter combines the positive feedback effect between abundance and occurrence, which could improve predictions and interpretations of patterns in biodiversity reacting to environmental change (Lindh et al., 2017). The core sub-communities are composed of the dominant species that are widely distributed and play a key role in the cycle of elements (Fuhrman, 2009; Pedrós-Alió, 2012), whereas the satellite sub-communities occur in low abundance and few locations and conduct specific metabolic functions, which constitute the seed bank of biodiversity (Pester et al., 2010; Van Der Gast et al., 2011; Lindh et al., 2017; Gendron et al., 2019). Up to now, this classification has proved to be a useful tool for understanding ecological principles of microorganisms, and has been applied in marine (Lindh et al., 2017) and rivers (Hu et al., 2017b) ecosystems, but has only infrequently been implemented in lake ecosystems.

Previous studies have reported that deterministic processes and stochastic processes play important roles in the regulation of spatial distribution of bacterial communities in natural environments (Sloan et al., 2006; Ofiţeru et al., 2010; Langenheder and Székely, 2011; Lindström and Östman, 2011; Lindström and Langenheder, 2012; Liao et al., 2016a,b). Deterministic processes refer to environmental filtering and biotic interactions influencing the fitness of microbial communities and determine the composition and abundance of microbes (Campbell et al., 2011; Gilbert et al., 2012; Zhang et al., 2014). Conversely, stochastic processes include dispersal limitation and random changes in species relative abundance, and therefore, changes in community composition are unpredictable (Hubbell, 2001; Dini-Andreote et al., 2015; Li et al., 2019). Recently, some studies have identified that different properties or traits of microbial sub-communities may assemble by different or same mechanisms (Pandit et al., 2009; Langenheder and Székely, 2011). For instance, the core and satellite sub-communities in a salinity-influenced watershed of China were mainly droved by deterministic processes (Hu et al., 2017b). The core sub-communities in arbuscular mycorrhizal fungi (AMF) were mainly influenced by deterministic processes related to soil properties, whereas the satellite sub-communities were considerably influenced by stochastic processes (Barnes et al., 2016). However, it remains unclear whether assembly processes of the core and satellite sub-communities in Tibetan lakes are similar or different when the range of distances over hundreds of kilometers? The ecological strategy can be elucidated by the contribution of deterministic and stochastic processes to microbial community assembly (Kraft et al., 2015; Jiao and Lu, 2020). Microorganisms with microscopic sizes and high dispersal capacity could display complex interaction webs within an ecological niche, which are also key to maintaining microbial community structure (Faust and Raes, 2012). Co-occurrence network analysis provides powerful support for revealing the complex microbial community structure and interactions among microorganisms, which could reflect shared niches among community members in the real world (Faust and Raes, 2012; Mikhailov et al., 2019; Mingkun et al., 2020). Hu et al. (2017b) demonstrated that due to different ecological niches, core and satellite sub-communities play different roles in the co-occurrence network and have different network topological characteristics.

In this study, we used 454 pyrosequencing of the bacterial 16S rRNA gene to investigate the diversity and composition of core and satellite bacterial sub-communities in 47 lake water samples of 30 lakes located on the Tibetan Plateau. The Tibetan Plateau has the largest number of plateau lakes group in the world (Zhang et al., 2011). A most recent study about the biogeography of microbial communities in Tibetan lakes reported that bacterial communities were mainly controlled by salinity-driven deterministic processes (Liu et al., 2020). Although the useful information gained from this study, the spatial distribution patterns, community assembly mechanisms, and the co-occurrence patterns may be different due to their different roles of the core and satellite bacterial sub-communities in the Tibetan lakes. Therefore, we sort to determine and compare the biogeographic patterns and underlying mechanisms for the core and satellite bacterial sub-communities at a regional scale. Specifically, we tested the following three hypotheses: (1) core and satellite taxa exhibit different biogeographic patterns in lakes on Tibetan Plateau; (2) core and satellite sub-communities assembly driven by divergent processes; and (3) compared to satellite, core sub-communities show a discrepant co-occurrence pattern.

## Materials and Methods

### Study Area and Sampling

We investigated surface water from 30 Tibetan lakes in 2012, China (Supplementary Figure S1). These lakes are characterized by high-altitude location (above 3,900 meters), which covered an area from 79.81'E to 96.82'E longitudinally and 28.27'N to 34.58'N latitudinally. The mean annual air temperature of the lakes ranged from −9°C to +2°C, and the surface area ranged from 8 to 2062km^2^ (Supplementary Table S1).

In total, 47 water samples were collected from 30 Tibetan lakes. The Schindler sampler was used to collect approximately 1L surface water samples (∼0.5m depth) from twenty lakes, respectively. Duplicate samples were collected at the same time from same points from 10 lakes, AGC, BC, BGC, DZC, GZC, NMC, PE, PMYC, Yamdrok, and ZGTC (Supplementary Table S1). Water samples from each site for bacterial community analyses were pre-filtered through 20μm mesh (Millipore, United States) for removal of the large plankton and particles, and all filtrates were subsequently filtered through a 0.22μm polycarbonate membrane (Millipore, united states). Afterward, the membranes were put in sterile 2ml microcentrifuge tubes and were stored at −80°C for DNA extraction. Latitude, longitude, and altitude were measured using the Global Positioning System during the field work.

### DNA Extraction, Bacterial 16S rRNA Amplification, and 454 Sequencing

Microbial DNA was extracted from filters using a FastDNA^®^ Spin kit (MP Biomedicals, Santa Ana, CA) according to the manufacturer’s instructions. It was checked for concentration and purity using a NanoDrop Spectrophotometer (ND-1000 Thermo Fisher Scientific, Wilmington, DE, United States). The V4-V5 region of the bacterial 16S rRNA genes was amplified using the primer pair 515F (5'-GTGCCAGCMGCCGCGGTAA-3') and 907R (5'-CCGTCAATTCMTTTRAGTTT-3'; Christopher et al., 2011). An aliquot of 10ng purified DNA template from each sample was amplified in triplicate in a 50μl reaction system. The amplification conditions were as follows: 30cycles of denaturation at 94°C for 30s, annealing at 55°C for 30s, and extension at 72°C for 30s, with a final extension at 72°C for 10min (Liu et al., 2019b). Then, triplicate PCR products for each sample were pooled in equal quantity and purified using agarose gel DNA purification kits (TaKaRa, Japan). Finally, running on a Roche FLX 454 pyrosequencing machine (Roche Diagnostics Corporation, Branford, CT, United States; Liu et al., 2016). Raw sequence reads have been submitted to NCBI (BioProject ID PRJNA306720).

### Processing of Pyrosequencing Data

Paired-end reads were quality trimmed using Trimmomatic v0.30 (Bolger et al., 2014) and combined using FLASH software (Magoč and Salzberg, 2011). The raw sequences data were subsequently analyzed by using QIIME v1.9.0 (Caporaso et al., 2010). The reads which had ambiguous bases and mismatches to the barcode or primers or chimeric characteristics were discarded. Then, the sequences were clustered into OTUs using UPARSE algorithm in USEARCH v 11.0.667 with a 97% threshold of sequence similarity (Edgar, 2013). Representative sequences of each OTU were aligned using PyNAST (DeSantis et al., 2006). Taxonomic identity of each phylotype was determined using the SILVA 132 database (Quast et al., 2013) via the RDP classifier (Wang et al., 2007). Before tree construction, the filter_alignment.py script in qiime1was used to remove highly variable regions, and then, a phylogenetic tree was constructed based on Neighbor-joining method (Saitou and Nei, 1987). All eukaryote, chloroplasts, mitochondria, and unknown sequences were culled before the OTU table was generated. To avoid biases generated by differences in sequencing depth and to make samples comparable, samples were randomly rarefied to the minimum number of retrieved sequences in the whole sample (2210). After taxonomies had been assigned, we deleted all archaea OTUs and obtained 5,233 OTUs and 103,870 sequences.

### Core and Satellite Sub-Community Classification

The Poisson model of species abundance was examined by Krebs’ method, and the dispersion index was tested by Chi-square test to partition the bacteria into the core and satellite sub-communities (Van Der Gast et al., 2011; Hu et al., 2017b). Bacterial taxa that occurred only in a single sample were excluded from this analysis because their distributed in space would have no variance. Briefly, OTU occurrence plotted against the index of dispersion (the ratio of variance to the mean abundance) for each OTU, taking 2.5% of the *χ*^2^ distribution as the confidence limit. OTUs that below the interval following a random distribution were considered as satellite sub-communities, whereas above were non-randomly distributed core sub-communities. Calculations were performed using the “vegan” and “plyr” R packages (Hu et al., 2017b).

### Distance Decay of the Community Dissimilarity

To evaluate the distance decay of community similarity, the linear regression between ln-transformed geographic distances and the Bray-Curtis dissimilarities was generated based on ordinary least squares. The relationships were evaluated using the Mantel test. The statistical significance of such comparisons was determined using 999 permutations and the analyses were performed using the “mantel” function of the “vegan” package in R (Jiao et al., 2020). Permutation test was used to test for significant differences between slopes in the “simba” R package (Nekola and White, 1999). Geographical distance between samples was calculated from the latitude and longitude coordinates using the “geosphere” packages (Hijmans, 2019). Bray-Curtis dissimilarities were based on the core and satellite OTU tables using the vegdist function in the “vegan” package (Zhu et al., 2019).

### Phylogenetic Null Model Analysis

Null model was used to quantify the contribution of different ecological processes (stochastic and deterministic; Stegen et al., 2013, 2015). This approach uses the beta mean nearest taxon distance (βMNTD) to represent the pairwise phylogenetic turnover between communities, and beta-nearest taxon index (βNTI) to represent the environmental impacts calculated by the standard deviation of the observed βMNTD from the βMNTD of the null model. When beta-nearest taxon index (βNTI)<−2 and≥2 was identified as homogeneous selection and heterogeneous selection, respectively. Moreover, 999 random permutations of communities generate a null distribution of Bray-Curtis dissimilarity, and a Raup-Crick metric (RC_bray_) is subsequently calculated by comparing empirically observed Bray-Curtis and simulated null distribution. The |βNTI|<2 and RC_bray_<−0.95 or the |βNTI|<2 and RC_bray_≥0.95 RCbray were identified as homogenizing dispersal and dispersal limitation, respectively. When the |βNTI|<2 and |RC_bray_|<0.95 were identified as “Undominated” (Dini-Andreote et al., 2015; Stegen et al., 2015; Isabwe et al., 2019). To demonstrate which process contributed more to the DDR slopes between the core and satellite sub-communities, samples controlled by dispersal limitation and heterogeneous selection were separately extracted from both sub-communities according to the results of Stegen’s null model. Then, the DDR slopes were calculated separately.

### Habitat Niche Breadth

Niche breadth is often used to identify different levels of habitat specialization, which is a crucial trait that affects the relative importance of selection and dispersal limitation affecting communities (Pandit et al., 2009; Logares et al., 2013; Liao et al., 2016a). Niche breadth was calculated using Levins’ niche breadth (Levins, 1968) index (*B*):

$$B_{j}=\frac{1}{\sum_{i=1}^{N}P_{ij}^{2}}$$

where *B_j_* represents the habitat niche breadth; *P_ij_* is the mean relative abundance of OTU *j* in lake *i* (i.e., one of the 30 water samples); and *N* is the total number of communities. A high B-values indicate a wide range of OTUs and even distribution, representing wide habitat niche breadth and more metabolic flexibility at the community level (Wu et al., 2018).

### Network Construction

We used network analysis to examine co-occurrence networks of core and satellite sub-communities. To reduce noise and complexity of the datasets, we kept OTUs that appeared in ≥5 samples for network analysis. Spearman’s rank coefficients (*ρ*) between those OTUs were calculated pairwise by the “Hmisc” package in an R environment. Only robust correlations with Spearman’s correlation coefficients (*ρ*)>0.6 and false discovery rate-corrected values of *p*<0.01 were used to construct networks (Hu et al., 2017a). Each node represents one OTU, and each edge represents a strong and significant correlation between two nodes. Network visualization was performed using the interactive platform Gephi (0.9.2). We use the “igraph” R package to calculate the node-level network topologies features (node degree, betweenness centrality, closeness centrality, and transitivity) and were examined by Kruskal-Wallis test to measure differences (Bastian et al., 2009; Mo et al., 2020). In addition, “igraph” package was used to calculate and compare the topology characteristics of the real networks and 10,000 Erdős-Rényi random networks, which had the same number of nodes and edges as the real networks (Jiao et al., 2020). To understand the stability of the core and satellite networks, two indices were used to characterize the stability, including robustness and vulnerability. Natural connections were used to assess network stability by removing nodes in the network to evaluate the rate of robustness degradation (Peng and Wu, 2016). Network vulnerability is expressed as the maximal vulnerability of nodes in the network (Yuan et al., 2021).

### Statistical Analyses

Diversity index was analyzed using “vegan” package in the R environment (R Core Team, 2013). Kruskal-Wallis test was performed with the PAST software to compare the α-diversity and niche differences of the core and satellite sub-communities and to identify the significantly and differentially abundant phyla/classes and genera between the core and satellite sub-communities. All the R analyses were performed in version 3.6.1.

## Results

### OTUs Composition and Diversity of the Core and Satellite Sub-Communities

**Figure 1:**
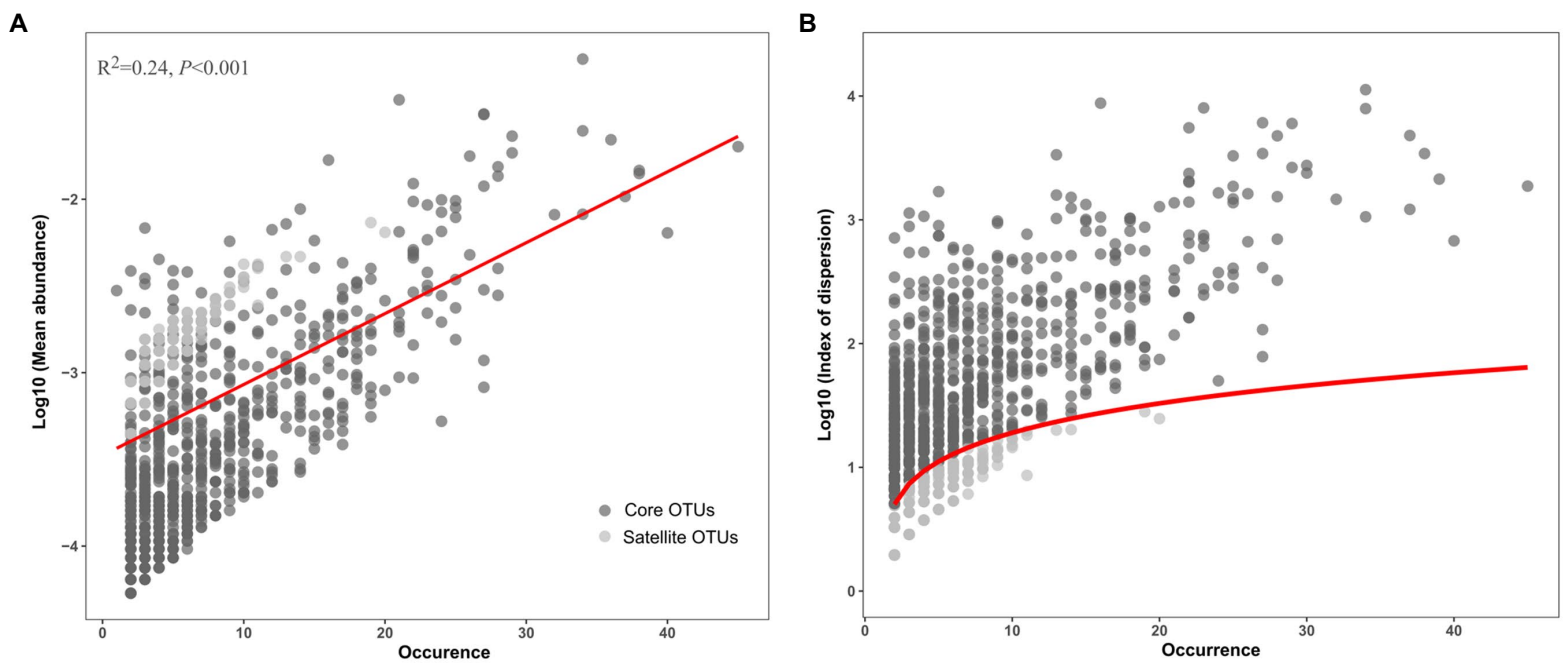
Distribution **(A)** and dispersion **(B)** of the core and satellite OTUs in the Tibetan lakes. **(A)** The OTU occurrence (number of samples in which a given OTU was detected) plotted against the mean relative abundance of each OTU across samples. The red line represents the linear regression model fit to species abundance distribution. **(B)** The OTU occurrence plotted against the index of dispersion for each OTU calculated as variance to mean ratio of abundance for each OTU. The red line represents 2.5% confidence limit for the *χ*^2^ distribution.

After removing low quality sequences, a total of 103,870 reads were obtained in this study and clustered into 5,233 OTUs (Table 1). Good’s coverage ranged from 86 to 96%, indicating that sequences identified in these samples represent the majority of bacterial sequences present in the collected water samples (Supplementary Table S2). A positive relationship between the mean abundance of OTUs and their occurrence was observed (*R*^2^=0.24, *p*<0.001; Figure 1A). The 1,276 OTUs fit a *χ*^2^ test were defined as the satellite sub-communities that with 4,500 (4.33%) reads. In contrast, the remaining 809 OTUs (93,493 reads), surpassing 2.5% confidence limit line of *χ*^2^ distribution, formed core sub-communities and accounted for 90.01% of the total reads (Table 1; Figure 1B).

**Table 1:** The number of OTUs and sequences of the core and satellite bacteria sub-communities.

Taxa	OTU number	Sequence number
ALL OTUs	5,233	103,870
Core OTUs	809 (15.46%)	93,493 (90.01%)
Satellite OTUs	1,276 (24.38%)	4,500 (4.33%)

**Figure 2:**
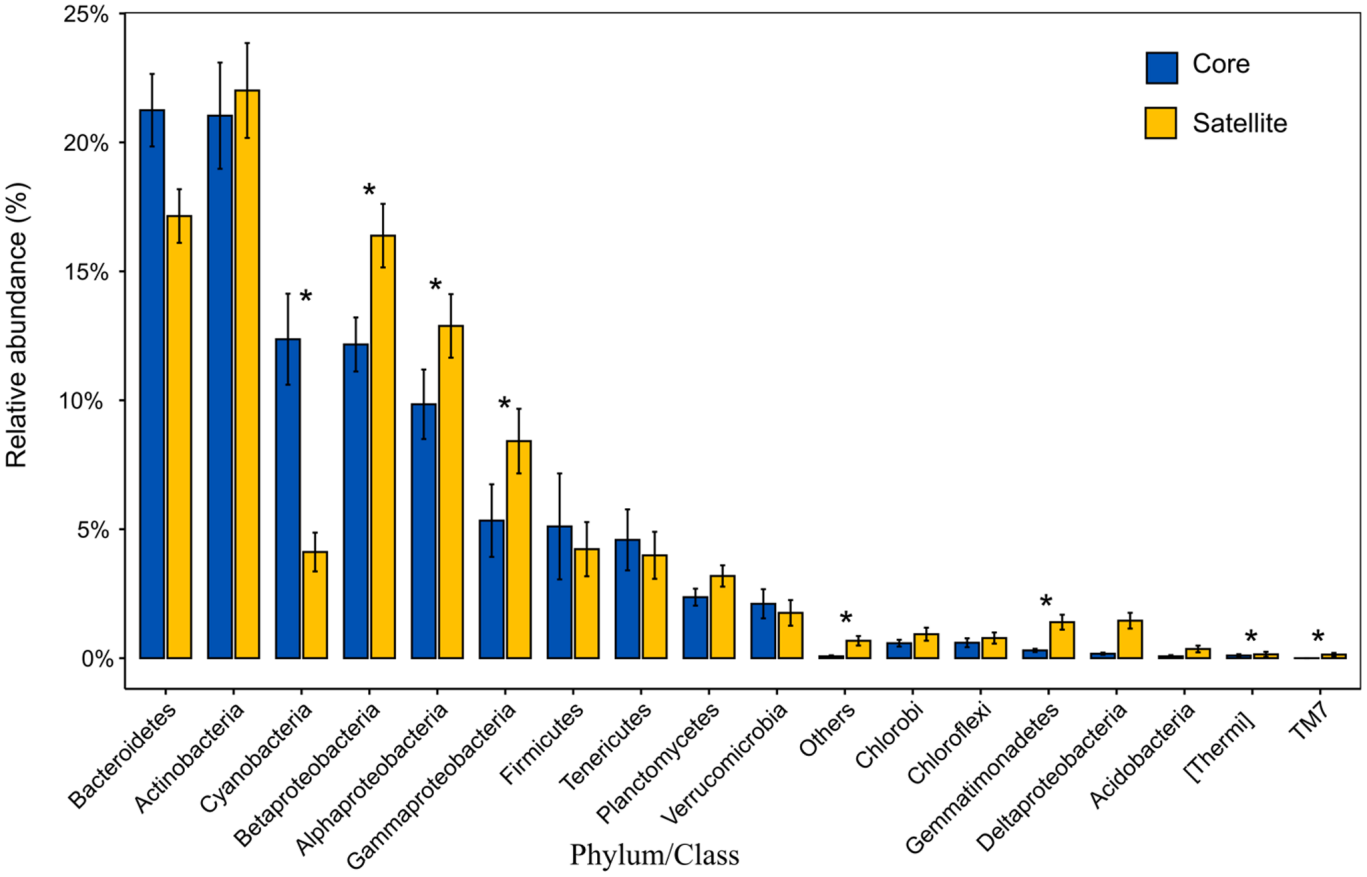
The relative abundance of the 18 common phyla and the classes of Proteobacteria within the core and satellite communities. Asterisks indicate significant differentially abundant phyla/class between the core and satellite bacterial sub-communities (*p*<0.05).

**Figure 3:**
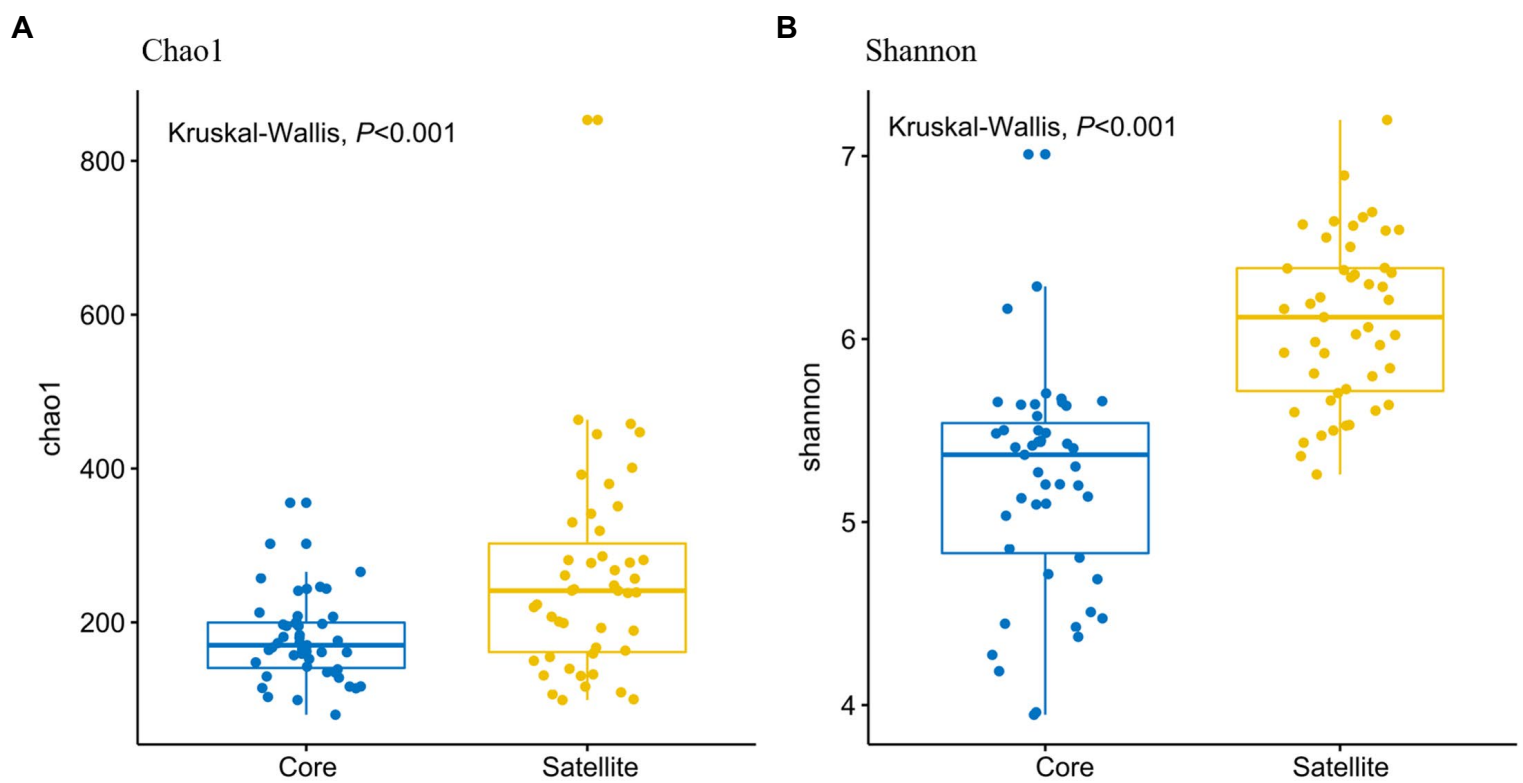
Comparison of α-diversities of the core and satellite bacterial sub-communities. **(A)** Chao1 index and **(B)** Shannon index.

In all taxa, Proteobacteria, Bacteroidetes, and Actinobacteria were the dominant phyla in the core and satellite sub-communities, together accounting for 71 and 78.62% of each sub-community sequences, respectively (Supplementary Table S3). Cyanobacteria was significantly abundant in the core sub-communities, while Betaproteobacteria, Alphaproteobacteria, Gammaproteobacteria, Gemmatimonadetes, Thermi, and TM7 were found to be significantly dominant in the satellite sub-communities (Kruskal-Wallis test, *p*<0.05; Figure 2). At the genus level, 43 genera showed significant differences between the two sub-communities (*p*<0.05; Supplementary Figure S2). Among them, the genera *Arthrobacter*, *B-42*, *Loktanella*, and *Rhodobacter* harbored a higher abundance in the satellite sub-communities, while some genera, such as *Planococcus*, *Psychroflexus*, and *Synechococcus* exhibited significantly higher abundances in the core sub-communities. The α-diversity indices of the core and satellite sub-communities were compared based on Chao1 and Shannon indices (Figure 3). Both Chao1 and Shannon indices of the satellite sub-communities were significantly higher than those of the core sub-communities (*p*<0.001).

### Geographic Patterns of the Core and Satellite Sub-Communities

**Figure 4:**
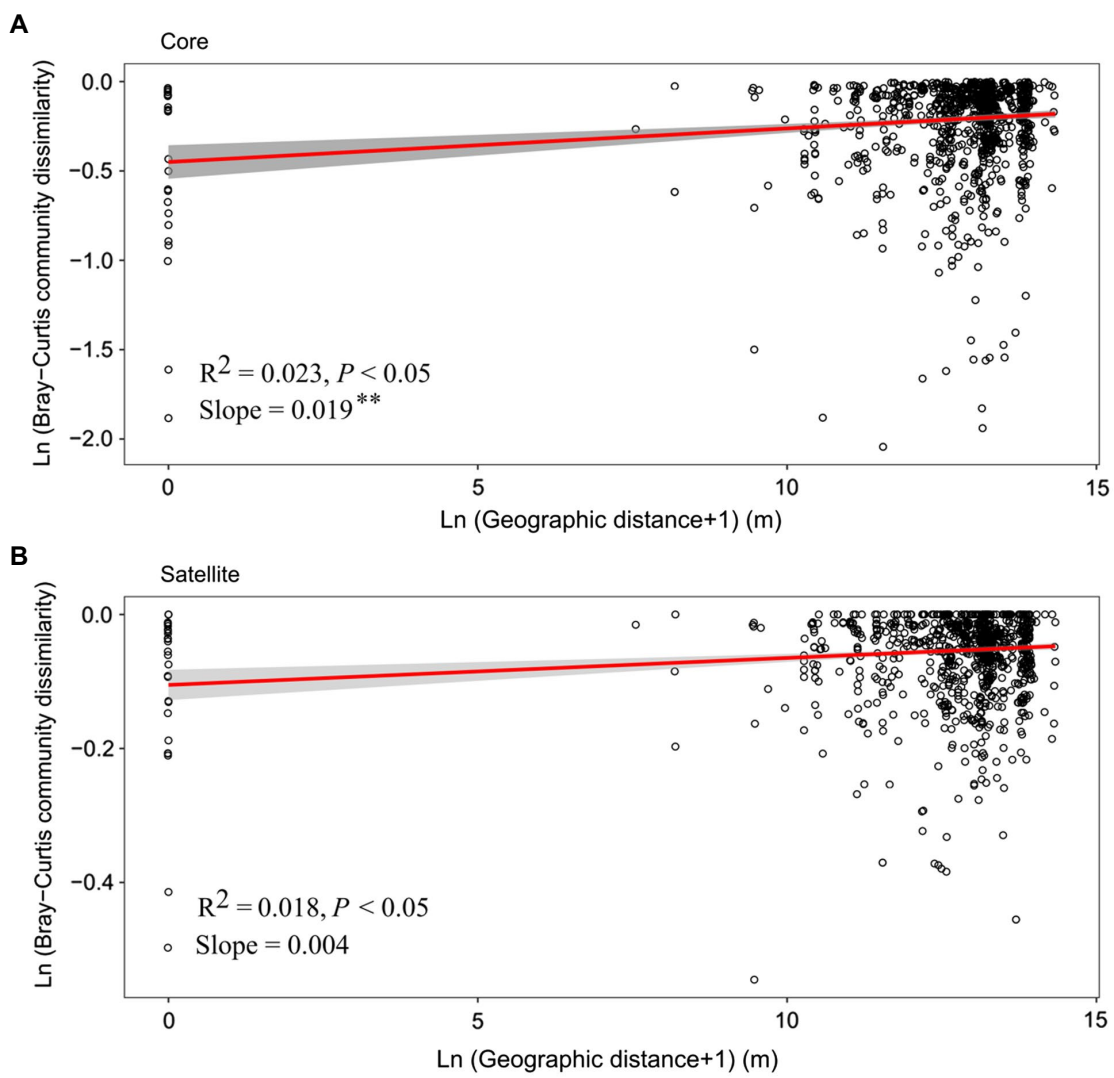
The relationship between geographic distances and Bray-Curtis dissimilarities of the **(A)** core and **(B)** satellite bacterial sub-communities. The red line in each plot represents a linear regression model fit to Ln (geographic distance+1) vs. Ln (Bray-Curtis community dissimilarity). Gray band around the line indicates 95% confidence interval. Asterisks denote significant different between slopes (*p*<0.01).

Distance-decay relationship (DDR) is a fundamental pattern in ecology, in which community similarity decreases as the geographic distance increases. In the current study, although the significant positive DDRs (Mantel *p*<0.05; Figure 4) were observed, the fitness values were relatively low (*R*^2^<0.1), indicating weak relationship of community dissimilarity with geographic distance for the core and satellite sub-communities. Meanwhile, the slope of DDRs was significant (*p*<0.01) steeper for the core sub-communities (0.019) than that of the satellite sub-communities (0.004).

### Niche Breadth and Ecological Processes Underlying the Core and Satellite Sub-Communities

The niche breadth (*B*) analysis indicated that the average niche breadth for the core communities (4.11) was significantly wider than that of the satellite communities (2.65; *p*<0.001; Supplementary Figure S3).

**Figure 5:**
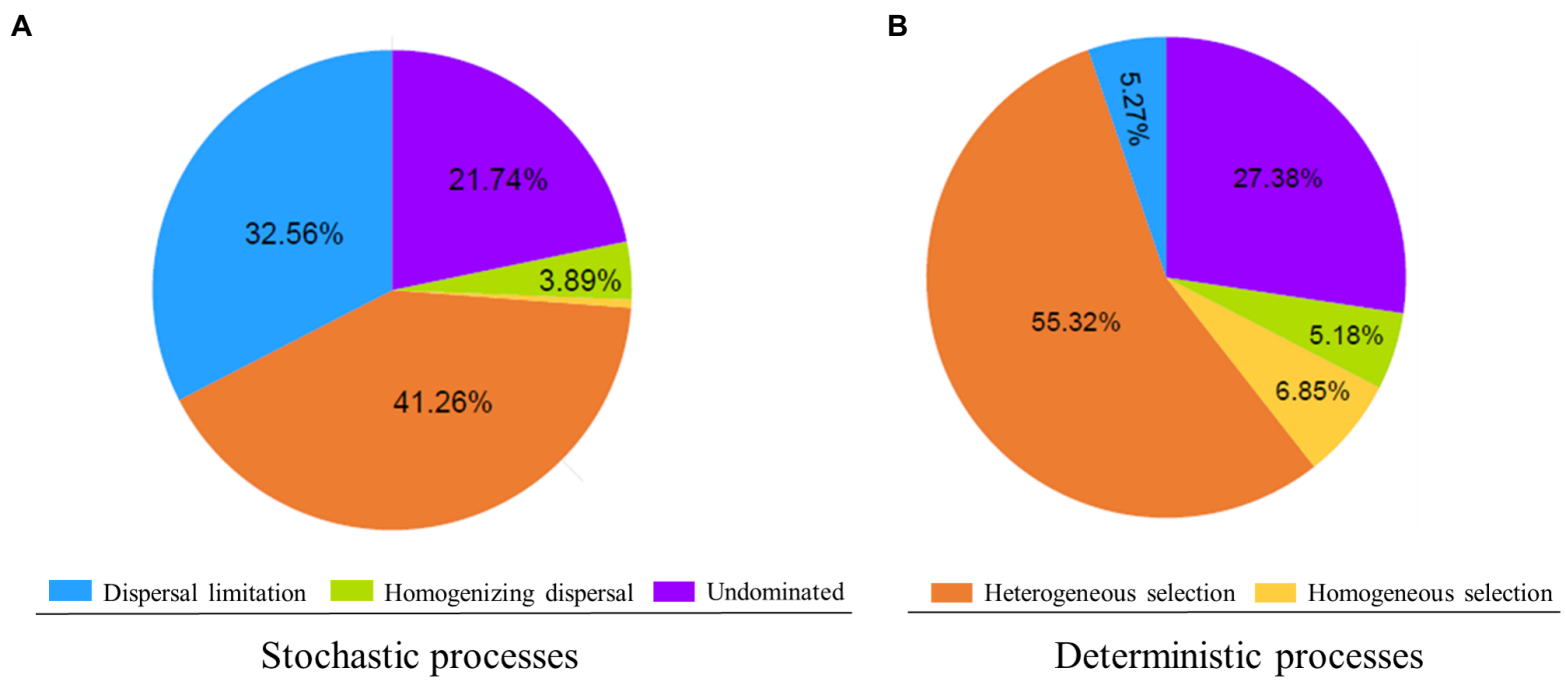
Delineation of the assembly processes underlying the core **(A)** and satellite **(B)** bacterial sub-communities. The percentage of turnovers governed by a process is used to represent its relative importance in community assembly. Low percentage contributions (<1.5%) are not shown.

The results of the null model quantify the relative contributions of major ecological processes of the core and satellite sub-communities in the Tibetan lakes (Figure 5). We found that heterogeneous selection was the most important process structuring of the core and satellite bacterial sub-communities (41.26 and 55.32% of the overall community turnover, respectively). Dispersal limitation and undominated showed similar relative importance in shaping the core sub-communities (32.56% vs. 21.74% of the turnover; Figure 5A). In contrast, undominated process contributed about 27.38% to shaping the satellite sub-communities, while that of dispersal limitation process was less than 5.5% (Figure 5B). Generally, the results recommended that stochastic processes explained a higher proportion of the core sub-community variations than deterministic processes, while satellite sub-communities were primarily affected by deterministic processes. As shown in Supplementary Figure S4, core sub-community turnover that controlled by dispersal limitation process showed a negative distance-decay slope (−0.004), while satellite sub-community turnover showed a slight positive distance-decay slope (0.001). On the contrary, core sub-community turnover governed by heterogeneous selection process was significantly higher (*p*<0.05) than that of satellite sub-communities (Supplementary Figure S4).

### Co-occurrence Network of the Core and Satellite Sub-Communities

**Figure 6:**
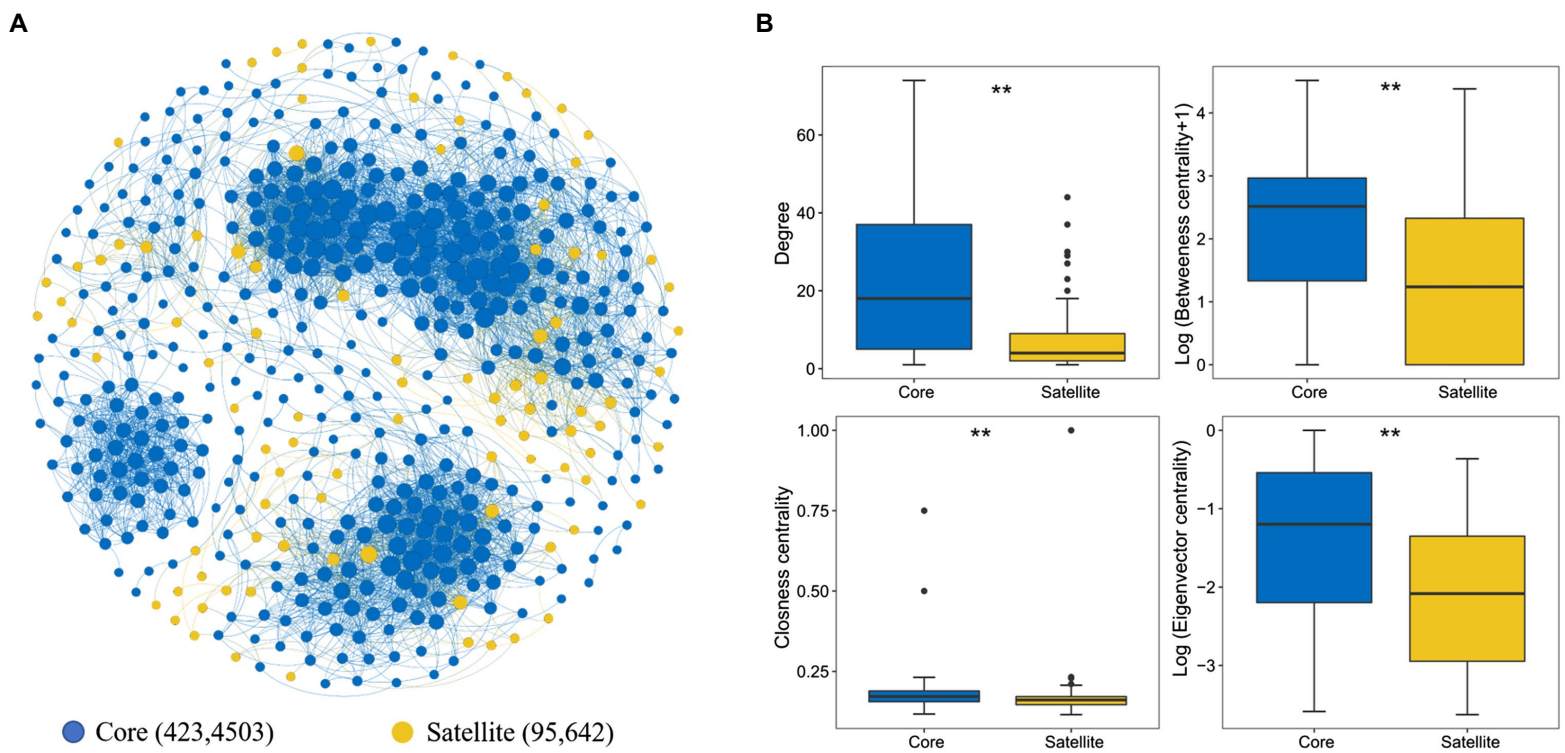
Properties of the correlation-based network among the core and satellite sub-communities. **(A)** Network of inter-taxon associations within and between core and satellite sub-communities. A connection stands for a strong (Spearman’s *ρ*>0.6 or *ρ*<−0.6) and significant (*p*<0.01) correlation. The size of each node is proportional to the degree of the OTU. Numbers represent the nodes and edges of the core and satellite sub-communities. **(B)**. Comparison of node-level topological features between two different sub-communities. “^**^” indicates significant differences (*p*<0.05), determined by the Kruskal-Wallis test.

The whole network included 5,145 associations among 518 microbial OTUs and exhibited scale-free characteristics (Power law: *R*^2^=0.71). Meanwhile, the real network exhibited higher values of average clustering coefficient (0.58 vs. 0.04), average path length (5.99 vs. 2.41), and modularity (0.64 vs. 0.19) than those of the respective Erdős-Rényi random, suggesting the real network was non-random and modular structure (Table 2). We identified 423 and 95 core and satellite OTUs throughout the whole network, respectively (Figure 6A). In addition, the degree, betweenness, closeness, and eigenvector showed significantly higher values in the core sub-communities bacterial co-occurrence patterns than that in the satellite sub-communities in the Tibetan lakes (*p* <0.01; Figure 6B). The core sub-communities co-occurrence network exhibited higher robustness structure and lower network vulnerability compared to the satellite sub-communities (Supplementary Figure S5), indicating that the core sub-community network was more stable.

**Table 2:** Topological properties of co-occurrence networks of the Tibetan lake bacterial communities and their corresponding random networks.

Network properties	Value
**Empirical network**	
Nodes	518
Edges	5,145
Average clustering coefficient	0.58
Diameter	9.73
Average path length	5.99
Average degree	19.86
Modularity	0.64
Power-law model	0.71
**Random networks**	
Average clustering coefficient	0.04±0.001
Average path length	2.41±0.001
Modularity	0.19±0.003

## Discussion

Community assembly mechanisms can predict community changes in space and time gradients, influence hydro-biogeochemical function, and have potential implications for ecosystem function and biodiversity conservation (Jiang and Patel, 2008; Hanson et al., 2012; Nemergut et al., 2013; Graham and Stegen, 2017). In this study, we used null model and network analysis to quantify the relative importance of ecological processes in shaping the core and satellite sub-communities and explore bacterial co-occurrence in the Tibetan lakes.

### Biogeographical Patterns of the Core and Satellite Communities

In this study, our results showed that both of the core and satellite bacterial sub-communities displayed significant DDRs (Mantel *p*<0.05; Figure 4). This implies that the core and satellite bacterial sub-communities were not a random collection of taxa (Liu et al., 2015). This was consistent with previous studies on freshwater lakes, reservoirs, and marine environments (Galand et al., 2009; Liu et al., 2015; Liao et al., 2017) and provided further evidence from Tibetan lakes. However, within this general pattern, we also observed that the DDR slope of the core sub-communities was steeper than that of the satellite sub-communities, suggesting that the spatial turnover rate of the core sub-communities is higher than the satellite counterparts. This finding is consistent with the research results on bacterial communities in the reservoirs and rivers (Liu et al., 2015). However, our results are opposite to an earlier study which revealed that the satellite taxonomic communities had higher spatial turnover rates than core counterparts in Yongjiang river watershed of China (Hu et al., 2017b). This contrary conclusion might be ascribed to the different research zones and habitat types. In Hu et al. (2017b)’s study, 29 river surface water were consideration. However, in the present study, 30 Tibetan lakes were studied, which exhibited larger geographic gradient.

### Ecological Processes Underlying the Assembly of the Core and Satellite Communities

Studies have shown that environmental filtering or dispersal-related processes can generate the DDRs of bacterial communities (Lindström and Östman, 2011; Liu et al., 2015). The process of environmental filtering generally differentiates microbial composition among locations, which will tend to produce a distance-decay relationship. By contrast, high dispersal will weak or eliminate the distance-decay relationship by counteracting compositional differentiation and the distance-decay relationship should be stronger when dispersal is more limited (Hanson et al., 2012). To identify the main reason underpinning the different DDR between the core and satellite sub-communities, we used a null model that did not involve spatial and explanatory variables. Our results suggest that the heterogeneous selection was the most important process in structuring the core and satellite sub-communities (41.26% vs. 55.32%). In heterogeneous selection, the slope of the core sub-communities exhibited significantly higher (*p*<0.05) than satellite sub-communities, while DDR controlled by dispersal limitation showed an opposite trend in the core and satellite sub-communities (Supplementary Figure S4). This could imply the important role of heterogeneous selection in shaping the different DDR slopes between the core and satellite sub-communities. A possible explanation for this might be due to environmental heterogeneity and the capability differences in the response to environmental change (Morrissey et al., 2019). Another possible explanation for this is that differences in species of the core and satellite sub-communities may form different cell size communities, generating the discrepant assembly mechanisms. Cell size has often been regarded as an important factor in affecting the metabolic versatility (Farjalla et al., 2012) and dispersal potential (Liu et al., 2019c) of organisms. The metabolic activities and dispersal abilities due to the effect of cell size may affect stochasticity or deterministic adequacy for explaining their community assembly (Zinger et al., 2019; Gao et al., 2020). Finally, the 21.74 and 27.38% undominated processes that contributed to the assembly of the core and sub-communities, indicating that these sub-communities were shape by a more complex assembly mechanism (Mo et al., 2018).

Bacterial sub-communities with wider niche breadth may have greater potential for dormancy (Wu et al., 2018; Mo et al., 2020). Thus, differences in niche breadth due to different species taxa and abundance in the core and satellite sub-communities (Supplementary Figure S2; Supplementary Table S3) can produce different dormancy strategies. The core sub-communities with wider niche breadth are more susceptible to enter dormancy of their cells than the satellite sub-communities, and reducing the active taxa affected by deterministic processes. This is an important metabolic strategy for microbial cells to manage with environmental stress and less vulnerable to deterministic processes (Lennon and Jones, 2011; Masanori et al., 2011; Nemergut et al., 2013).

### Co-existence Patterns of the Core and Satellite Communities

Co-occurrence networks can partially reveal complex interactions within microbial communities and have been considered to be an important tools for investigating potential interactions within microbial food webs (Faust and Raes, 2012; Liu et al., 2019a; Du et al., 2020). Network topology features can reflect the complex interactions between microorganisms in the community. The present study showed that the core sub-communities have significantly higher network topology than satellite (Figure 6B). This suggests that there are stronger and more complex webs of interaction in the core than in the satellite sub-communities. Specific properties promoted community stability in co-occurrence networks, and competition could also increase the stability of the community structure (Ghoul and Mitri, 2016). More complex network structure indicates stronger connections between competitors and more efficient resource transfer (Morriën et al., 2017; Yao et al., 2019). The core sub-community network had higher connectivity than satellite networks (Supplementary Figure S5A), which suggests that it was more efficient at transferring information, energy, and resources. On the other hand, the simple network structure also reflects the fragility of the satellite bacterial sub-community structure in the case of ecosystem perturbations (Wang et al., 2018). In addition, our study also supports to Ghoul and Mitri’s (2016) argument that increasing the complexity of a co-occurrence network leads to more stable co-existence patterns.

## Conclusion

In summary, this study has provided a better understanding of assembly mechanisms and co-occurrence patterns of the core and satellite bacterial sub-communities across multiple Tibetan lakes. Our results demonstrated that the core bacterial sub-communities exhibited similar biogeographic patterns to the satellite counterparts, but their patterns were generally shaped via different assembly mechanisms. For the core sub-communities, stochastic processes played important roles, while deterministic processes are of importance in shaping the satellite sub-community assembly. The co-occurrence pattern of the core sub-communities was more complex and more stable. Therefore, in future studies, bacterial community should be distinguished by traits of taxa in order to obtain comprehensive understanding of the biogeography and co-occurrence patterns of lake bacterial community.

Although the ecological model used can provide the in-depth results on the community assembly mechanisms, we acknowledge some limitations in the study. For example, the null model relies more on phylogenetic tree and lacks an explanation of the results through environmental factors. Therefore, it is necessary to use the null model and environment factors analysis at the same time in the subsequent research in order to obtain richer conclusions.

## Data Availability Statement

The datasets presented in this study can be found in online repositories. The names of the repository/repositories and accession number(s) can be found at NCBI (BioProject ID PRJNA306720).

## Author Contributions

This work was conceived by KL and YL. Field work was done by KL. Laboratory work was done by KL and FW. Analysis was carried out and the manuscript was written by QY and KL. Writing-reviewing and editing were done by JD. All authors contributed to the article and approved the submitted version.

## Funding

This work was supported by the National Natural Science Foundation of China (grant nos. 41771086 and 42006200), the Second Tibetan Plateau Scientific Expedition and Research Program (STEP; grant no. 2019QZKK0503), the National Key Research and Development Program Project (grant no. 2019YFC1509103), and the Strategic Priority Research Program (A) of the Chinese Academy of Sciences (grant no. XDA20050101).

## Conflict of Interest

The authors declare that the research was conducted in the absence of any commercial or financial relationships that could be construed as a potential conflict of interest.

## Publisher’s Note

All claims expressed in this article are solely those of the authors and do not necessarily represent those of their affiliated organizations, or those of the publisher, the editors and the reviewers. Any product that may be evaluated in this article, or claim that may be made by its manufacturer, is not guaranteed or endorsed by the publisher.

## References

[ref1] Alonso-Sáez L. Díaz-Pérez L. Morán X. A. G. (2015). The hidden seasonality of the rare biosphere in coastal marine bacterioplankton. Environ. Microbiol. 17, 3766–3780. doi: 10.1111/1462-2920.12801, PMID: 25684402

[ref2] Barnes C. J. Burns C. A. Van D. G. C. J. Mcnamara N. P. Bending G. D. (2016). Spatio-temporal variation of core and satellite arbuscular mycorrhizal fungus communities in miscanthus giganteus. Front. Microbiol. 7:1278. doi: 10.3389/fmicb.2016.01278, PMID: 27597844 PMC4993019

[ref3] Bastian M. Heymann S. Jacomy M. (2009). Gephi: an open source software for exploring and manipulating networks. ICWSM 8, 361–362. doi: 10.13140/2.1.1341.1520

[ref4] Bolger A. M. Marc L. Bjoern U. (2014). Trimmomatic: a flexible trimmer for Illumina sequence data. Bioinformatics 15, 2114–2120. doi: 10.1093/bioinformatics/btu170, PMID: 24695404 PMC4103590

[ref5] Campbell B. J. Yu L. Heidelberg J. F. Kirchman D. L. (2011). Activity of abundant and rare bacteria in a coastal ocean. Proc. Natl. Acad. Sci. U. S. A. 108, 12776–12781. doi: 10.1073/pnas.1101405108, PMID: 21768380 PMC3150899

[ref6] Caporaso J. G. Kuczynski J. Stombaugh J. Bittinger K. Bushman F. D. Costello E. K.. (2010). QIIME allows analysis of high-throughput community sequencing data. Nat. Methods 7, 335–336. doi: 10.1038/nmeth.f.303, PMID: 20383131 PMC3156573

[ref7] Christopher Q. Anders L. Russell D. P.. (2011). Removing noise from pyrosequenced amplicons. BMC Bioinf. 12:38. doi: 10.1186/1471-2105-12-38, PMID: 21276213 PMC3045300

[ref8] DeSantis T. Z. Hugenholtz P. Keller K. Brodie E. L. Larsen N. Piceno Y. M.. (2006). NAST: a multiple sequence alignment server for comparative analysis of 16s rRNA genes. Nucleic Acids Res. 34, 394–399. doi: 10.1093/nar/gkl244, PMID: 16845035 PMC1538769

[ref9] Dini-Andreote F. Stegen J. C. van Elsas J. D. Salles J. F. (2015). Disentangling mechanisms that mediate the balance between stochastic and deterministic processes in microbial succession. Proc. Natl. Acad. Sci. U. S. A. 112, E1326–E1332. doi: 10.1073/pnas.1414261112, PMID: 25733885 PMC4371938

[ref10] Du S. Dini-Andreote F. Zhang N. Liang C. Yao Z. Zhang H.. (2020). Divergent co-occurrence patterns and assembly processes structure the abundant and rare bacterial communities in a salt marsh ecosystem. Appl. Environ. Microbiol. 86:e00322. doi: 10.1128/AEM.00322-20, PMID: 32358000 PMC7301849

[ref11] Edgar R. C. (2013). UPARSE: highly accurate OTU sequences from microbial amplicon reads. Nat. Methods 10, 996–998. doi: 10.1038/nmeth.2604, PMID: 23955772

[ref12] Farjalla V. F. Srivastava D. S. Marino N. A. Azevedo F. D. Dib V. Lopes P. M.. (2012). Ecological determinism increases with organism size. Ecology 93, 1752–1759. doi: 10.1890/11-1144.1, PMID: 22919920

[ref13] Faust K. Raes J. (2012). Microbial interactions: from networks to models. Nat. Rev. Microbiol. 10, 538–550. doi: 10.1038/nrmicro2832, PMID: 22796884

[ref14] Fuhrman J. A. (2009). Microbial community structure and its functional implications. Nature 459, 193–199. doi: 10.1038/nature08058, PMID: 19444205

[ref15] Galand P. E. Casamayor E. O. Kirchman D. L. Lovejoy C. (2009). Ecology of the rare microbial biosphere of the Arctic Ocean. Proc. Natl. Acad. Sci. U. S. A. 106, 22427–22432. doi: 10.1073/pnas.0908284106, PMID: 20018741 PMC2796907

[ref16] Gao C. Montoya L. Xu L. Madera M. Hollingsworth J. Purdom E.. (2020). Fungal community assembly in drought-stressed sorghum shows stochasticity, selection, and universal ecological dynamics. Nat. Commun. 11:34. doi: 10.1038/s41467-019-13913-9, PMID: 31911594 PMC6946711

[ref17] Gendron E. M. S. Darcy J. L. Hell K. Schmidt S. K. (2019). Structure of bacterial and eukaryote communities reflect in situ controls on community assembly in a high-alpine lake. J. Microbiol. 57, 852–864. doi: 10.1007/s12275-019-8668-8, PMID: 31376109

[ref18] Ghoul M. Mitri S. (2016). The ecology and evolution of microbial competition. Trends Microbiol. 24, 833–845. doi: 10.1016/j.tim.2016.06.011, PMID: 27546832

[ref19] Gilbert J. A. Steele J. A. Caporaso J. G. Steinbrück L. Reeder J. Temperton B.. (2012). Defining seasonal marine microbial community dynamics. ISME J. 6, 298–308. doi: 10.1038/ismej.2011.107, PMID: 21850055 PMC3260500

[ref20] Graham E. B. Stegen J. C. (2017). Dispersal-based microbial community assembly decreases biogeochemical function. PRO 5:65. doi: 10.3390/pr5040065

[ref21] Hanson C. A. Fuhrman J. A. Horner-Devine M. C. Martiny J. B. H. (2012). Beyond biogeographic patterns: processes shaping the microbial landscape. Nat. Rev. Microbiol. 10, 497–506. doi: 10.1038/nrmicro2795, PMID: 22580365

[ref22] Hijmans R. J. (2019). Introduction to the “geosphere” package (Version 1.5-10).

[ref23] Hu A. Ju F. Hou L. Li J. Yang X. Wang H.. (2017a). Strong impact of anthropogenic contamination on the co-occurrence patterns of a riverine microbial community. Environ. Microbiol. 19, 4993–5009. doi: 10.1111/1462-2920.13942, PMID: 28967165

[ref24] Hu A. Wang H. Yang X. Hou L. Li J. Li S.. (2017b). Seasonal and spatial variations of prokaryoplankton communities in a salinity-influenced watershed, China. FEMS Microbiol. Ecol. 93:fix093. doi: 10.1093/femsec/fix093, PMID: 28810707

[ref25] Hubbell S. P. (2001). The Unified Neutral Theory of Biodiversity and Biogeography (MPB-32). Princeton, New Jersey: Princeton University Press.

[ref26] Isabwe A. Ren K. Wang Y. Peng F. Chen H. Yang J. (2019). Community assembly mechanisms underlying the core and random bacterioplankton and microeukaryotes in a river–reservoir system. Water 11:1127. doi: 10.3390/w11061127

[ref27] Jeanbille M. Gury J. Duran R. Tronczynski J. Agogué H. Ben Saïd O.. (2016). Response of core microbial consortia to chronic hydrocarbon contaminations in coastal sediment habitats. Front. Microbiol. 7:1637. doi: 10.3389/fmicb.2016.01637, PMID: 27790213 PMC5061854

[ref28] Jiang L. Patel S. N. (2008). Community assembly in the presence of disturbance: a microcosm experiment. Ecology 89, 1931–1940. doi: 10.1890/07-1263.1, PMID: 18705379

[ref29] Jiao S. Lu Y. (2020). Soil pH and temperature regulate assembly processes of abundant and rare bacterial communities in agricultural ecosystems. Environ. Microbiol. 22, 1052–1065. doi: 10.1111/1462-2920.14815, PMID: 31599105

[ref30] Jiao S. Yang Y. Xu Y. Zhang J. Lu Y. (2020). Balance between community assembly processes mediates species coexistence in agricultural soil microbiomes across eastern China. ISME J. 14, 202–216. doi: 10.1038/s41396-019-0522-9, PMID: 31611655 PMC6908645

[ref31] Kraft N. J. Adler P. B. Godoy O. James E. C. Fuller S. Levine J. M. (2015). Community assembly, coexistence and the environmental filtering metaphor. Funct. Ecol. 29, 592–599. doi: 10.1111/1365-2435.12345

[ref32] Langenheder S. Székely A. J. (2011). Species sorting and neutral processes are both important during the initial assembly of bacterial communities. ISME J. 5, 1086–1094. doi: 10.1038/ismej.2010.207, PMID: 21270841 PMC3146284

[ref33] Lennon J. T. Jones S. E. (2011). Microbial seed banks: the ecological and evolutionary implications of dormancy. Nat. Rev. Microbiol. 9, 119–130. doi: 10.1038/nrmicro2504, PMID: 21233850

[ref34] Levins R. (1968). Evolution in Changing Environments: Some Theoretical Explorations (MPB-2). Princeton, New Jersey: Princeton University Press.

[ref35] Li Y. Gao Y. Zhang W. Wang C. Wu H. (2019). Homogeneous selection dominates the microbial community assembly in the sediment of the three gorges reservoir. Sci. Total Environ. 690, 50–60. doi: 10.1016/j.scitotenv.2019.07.014 31284194

[ref36] Liao J. Cao X. Wang J. Zhao L. Sun J. Jiang D.. (2017). Similar community assembly mechanisms underlie similar biogeography of rare and abundant bacteria in lakes on Yungui Plateau, China. Limnol. Oceanogr. 62, 723–735. doi: 10.1002/lno.10455

[ref37] Liao J. Cao X. Zhao L. Wang J. Gao Z. Wang M. C.. (2016a). The importance of neutral and niche processes for bacterial community assembly differs between habitat generalists and specialists. FEMS Microbiol. Ecol. 92:fiw174. doi: 10.1093/femsec/fiw174, PMID: 27543321

[ref38] Liao J. Zhao L. Cao X. Sun J. Gao Z. Wang J.. (2016b). Cyanobacteria in lakes on Yungui Plateau, China are assembled via niche processes driven by water physicochemical property, lake morphology and watershed land-use. Sci. Rep. 6:36357. doi: 10.1038/srep36357, PMID: 27819304 PMC5098255

[ref39] Lindh M. V. Sjöstedt J. Ekstam B. Casini M. Lundin D. Hugerth L. W.. (2017). Metapopulation theory identifies biogeographical patterns among core and satellite marine bacteria scaling from tens to thousands of kilometers. Environ. Microbiol. 19, 1222–1236. doi: 10.1111/1462-2920.13650, PMID: 28028880

[ref40] Lindström E. S. Langenheder S. (2012). Local and regional factors influencing bacterial community assembly. Environ. Microbiol. Rep. 4, 1–9. doi: 10.1111/j.1758-2229.2011.00257.x, PMID: 23757223

[ref41] Lindström E. S. Östman Ö. (2011). The importance of dispersal for bacterial community composition and functioning. PLoS One 6:e25883. doi: 10.1371/journal.pone.0025883, PMID: 21998714 PMC3188564

[ref42] Liu K. Hou J. Liu Y. Hu A. Wang M. Wang F.. (2019c). Biogeography of the free-living and particle-attached bacteria in Tibetan lakes. FEMS Microbiol. Ecol. 95:fiz088. doi: 10.1093/femsec/fiz088, PMID: 31183497

[ref001] Liu K. Liu Y. Hu A. Wang F. Chen Y. Gu Z.. (2020). Different community assembly mechanisms underlie similar biogeography of bacteria and microeukaryotes in Tibetan lakes. FEMS microbiol. ecol. 96:fiaa071., PMID: 32310264 10.1093/femsec/fiaa071

[ref43] Liu K. Liu Y. Jiao N. Zhu L. Wang J. Hu A.. (2016). Vertical variation of bacterial community in Nam Co, a large stratified lake in central Tibetan Plateau. Antonie Van Leeuwenhoek 109, 1323–1335. doi: 10.1007/s10482-016-0731-4, PMID: 27406261

[ref44] Liu J. Meng Z. Liu X. Zhang X.-H. (2019a). Microbial assembly, interaction, functioning, activity and diversification: a review derived from community compositional data. Mar. Life Sci. Technol. 1, 1–17. doi: 10.1007/s42995-019-00004-3

[ref45] Liu L. Yang J. Yu Z. Wilkinson D. M. (2015). The biogeography of abundant and rare bacterioplankton in the lakes and reservoirs of China. ISME J. 9, 2068–2077. doi: 10.1038/ismej.2015.29, PMID: 25748371 PMC4542038

[ref46] Liu K. Yao T. Liu Y. Xu B. Hu A. Chen Y. (2019b). Elevational patterns of abundant and rare bacterial diversity and composition in mountain streams in the southeast of the Tibetan Plateau. Sci. Earth Sci. 62, 853–862. doi: 10.1007/s11430-018-9316-6

[ref47] Logares R. Lindström E. S. Langenheder S. Logue J. B. Paterson H. Laybourn-Parry J.. (2013). Biogeography of bacterial communities exposed to progressive long-term environmental change. ISME J. 7, 937–948. doi: 10.1038/ismej.2012.168, PMID: 23254515 PMC3635229

[ref48] Magoč T. Salzberg S. L. (2011). FLASH: fast length adjustment of short reads to improve genome assemblies. Bioinformatics 27, 2957–2963. doi: 10.1093/bioinformatics/btr507, PMID: 21903629 PMC3198573

[ref49] Magurran A. E. Henderson P. A. (2003). Explaining the excess of rare species in natural species abundance distributions. Nature 422, 714–716. doi: 10.1038/nature01547, PMID: 12700760

[ref50] Masanori F. Hisaya K. Tomoya I.. (2011). Dissolved organic carbon as major environmental factor affecting bacterioplankton communities in mountain lakes of eastern Japan. Microb. Ecol. 63, 496–508. doi: 10.1007/s00248-011-9983-8, PMID: 22109097

[ref51] Mikhailov I. S. Zakharova Y. R. Bukin Y. S. Galachyants Y. P. Petrova D. P. Sakirko M. V.. (2019). Co-occurrence networks among bacteria and microbial eukaryotes of lake baikal during a spring phytoplankton bloom. Microb. Ecol. 77, 96–109. doi: 10.1007/s00248-018-1212-2, PMID: 29882155

[ref52] Mingkun L. Xue H. Jun T. Huifeng Z. Xiaohui B. (2020). Mutual environmental drivers of the community composition, functional attributes and co-occurrence patterns of bacterioplankton in the composite aquatic ecosystem of Taihu watershed in China. FEMS Microbiol. Ecol. 8:fiaa137. doi: 10.1093/femsec/fiaa137, PMID: 32639543

[ref53] Mo Y. Zhang W. Wilkinson D. M. Yu Z. Xiao P. Yang J. (2020). Biogeography and co-occurrence patterns of bacterial generalists and specialists in three subtropical marine bays. Limnol. Oceanogr. 66, 793–508. doi: 10.1002/lno.11643

[ref54] Mo Y. Zhang W. Yang J. Lin Y. Yu Z. Lin S. (2018). Biogeographic patterns of abundant and rare bacterioplankton in three subtropical bays resulting from selective and neutral processes. ISME J. 12, 2198–2210. doi: 10.1038/s41396-018-0153-6, PMID: 29880912 PMC6092436

[ref55] Morriën E. Hannula S. E. Snoek L. B. Helmsing N. R. Zweers H. De Hollander M.. (2017). Soil networks become more connected and take up more carbon as nature restoration progresses. Nat. Commun. 8:14349. doi: 10.1038/ncomms14349, PMID: 28176768 PMC5309817

[ref56] Morrissey E. M. Mau R. L. Hayer M. Liu X.-J. Schwartz E. Dijkstra P.. (2019). Evolutionary history constrains microbial traits across environmental variation. Nat. Ecol. Evol. 3, 1064–1069. doi: 10.1038/s41559-019-0918-y, PMID: 31209289

[ref57] Nekola J. C. White P. S. (1999). The distance decay of similarity in biogeography and ecology. J. Biogeogr. 26, 867–878. doi: 10.1046/j.1365-2699.1999.00305.x

[ref58] Nemergut D. R. Schmidt S. K. Fukami T. O’Neill S. P. Bilinski T. M. Stanish L. F.. (2013). Patterns and processes of microbial community assembly. Microbiol. Mol. Biol. Rev. 77, 342–356. doi: 10.1128/MMBR.00051-12, PMID: 24006468 PMC3811611

[ref59] Ofiţeru I. D. Lunn M. Curtis T. P. Wells G. F. Criddle C. S. Francis C. A.. (2010). Combined niche and neutral effects in a microbial wastewater treatment community. Proc. Natl. Acad. Sci. U. S. A. 107, 15345–15350. doi: 10.1073/pnas.1000604107, PMID: 20705897 PMC2932620

[ref60] Pandit S. N. Kolasa J. Cottenie K. (2009). Contrasts between habitat generalists and specialists: an empirical extension to the basic metacommunity framework. Ecology 90, 2253–2262. doi: 10.1890/08-0851.1, PMID: 19739387

[ref61] Pedrós-Alió C. (2012). The rare bacterial biosphere. Annu. Rev. Mar. Sci. 4, 449–466. doi: 10.1146/annurev-marine-120710-100948, PMID: 22457983

[ref62] Peng G. S. Wu J. (2016). Optimal network topology for structural robustness based on natural connectivity. Physica A 443, 212–220. doi: 10.1016/j.physa.2015.09.023

[ref63] Pester M. Bittner N. Deevong P. Wagner M. Loy A. (2010). A ‘rare biosphere’microorganism contributes to sulfate reduction in a peatland. ISME J. 4, 1591–1602. doi: 10.1038/ismej.2010.75, PMID: 20535221 PMC4499578

[ref64] Quast C. Pruesse E. Yilmaz P. Gerken J. Schweer T. Yarza P.. (2013). The SILVA ribosomal RNA gene database project: improved data processing and web-based tools. Nucleic Acids Res. 41, D590–D596. doi: 10.1093/nar/gks1219, PMID: 23193283 PMC3531112

[ref65] R Core Team (2013) R: A Language and Environment for Statistical Computing. Vienna, Austria: R Foundation for Statistical Computing.

[ref66] Saitou N. Nei M. (1987). The neighbor-joining method: a new method for reconstructing phylogenetic trees. Mol. Biol. Evol. 4, 406–425. doi: 10.1093/oxfordjournals.molbev.a040454, PMID: 3447015

[ref67] Sloan W. T. Lunn M. Woodcock S. Head I. M. Nee S. Curtis T. P. (2006). Quantifying the roles of immigration and chance in shaping prokaryote community structure. Environ. Microbiol. 8, 732–740. doi: 10.1111/j.1462-2920.2005.00956.x, PMID: 16584484

[ref68] Stegen J. C. Lin X. Fredrickson J. K. Chen X. Kennedy D. W. Murray C. J.. (2013). Quantifying community assembly processes and identifying features that impose them. ISME J. 7, 2069–2079. doi: 10.1038/ismej.2013.93, PMID: 23739053 PMC3806266

[ref69] Stegen J. C. Lin X. Fredrickson J. K. Konopka A. E. (2015). Estimating and mapping ecological processes influencing microbial community assembly. Front. Microbiol. 6:370. doi: 10.3389/fmicb.2015.00370, PMID: 25983725 PMC4416444

[ref71] Unterseher M. Jumpponen A. Oepik M. Tedersoo L. Moora M. Dormann C. F.. (2011). Species abundance distributions and richness estimations in fungal metagenomics–lessons learned from community ecology. Mol. Ecol. 20, 275–285. doi: 10.1111/j.1365-294X.2010.04948.x, PMID: 21155911

[ref72] Van Der Gast C. J. Walker A. W. Stressmann F. A. Rogers G. B. Scott P. Daniels T. W.. (2011). Partitioning core and satellite taxa from within cystic fibrosis lung bacterial communities. ISME J. 5, 780–791. doi: 10.1038/ismej.2010.175, PMID: 21151003 PMC3105771

[ref73] Wang Q. Garrity G. M. Tiedje J. M. Cole J. R. (2007). Naive Bayesian classifier for rapid assignment of rRNA sequences into the new bacterial taxonomy. Appl. Environ. Microbiol. 73, 5261–5267. doi: 10.1128/AEM.00062-07, PMID: 17586664 PMC1950982

[ref74] Wang S. Wang X. Han X. Deng Y. (2018). Higher precipitation strengthens the microbial interactions in semi-arid grassland soils. Global Ecol. Biogeogr. 27, 570–580. doi: 10.1111/geb.12718

[ref75] Wu W. Lu H.-P. Sastri A. Yeh Y.-C. Gong G.-C. Chou W.-C.. (2018). Contrasting the relative importance of species sorting and dispersal limitation in shaping marine bacterial versus protist communities. ISME J. 12, 485–494. doi: 10.1038/ismej.2017.183, PMID: 29125596 PMC5776463

[ref76] Yao Z. Du S. Liang C. Zhao Y. Dini-Andreote F. Wang K.. (2019). Bacterial community assembly in a typical estuarine marsh with multiple environmental gradients. Appl. Environ. Microbiol. 85:e02602. doi: 10.1128/AEM.02602-18, PMID: 30635381 PMC6414364

[ref77] Yuan M. M. Guo X. Wu L. Zhang Y. Xiao N. Ning D.. (2021). Climate warming enhances microbial network complexity and stability. Nat. Clim. Chang. 11, 343–348. doi: 10.1038/s41558-021-00989-9

[ref78] Zhang G. Xie H. Kang S. Yi D. Ackley S. F. (2011). Monitoring lake level changes on the Tibetan Plateau using ICESat altimetry data (2003–2009). Remote Sens. Environ. 115, 1733–1742. doi: 10.1016/j.rse.2011.03.005

[ref79] Zhang Y. Zhao Z. Dai M. Jiao N. Herndl G. J. (2014). Drivers shaping the diversity and biogeography of total and active bacterial communities in the South China Sea. Mol. Ecol. 23, 2260–2274. doi: 10.1111/mec.12739, PMID: 24684298 PMC4230472

[ref80] Zhu H. Z. Zhang Z. F. Zhou N. Jiang C. Y. Wang B. J. Cai L.. (2019). Diversity, distribution and co-occurrence patterns of bacterial communities in a karst cave system. Front. Microbiol. 10:1726. doi: 10.3389/fmicb.2019.01726, PMID: 31447801 PMC6691740

[ref81] Zinger L. Taberlet P. Schimann H. Bonin A. Boyer F. De Barba M.. (2019). Body size determines soil community assembly in a tropical forest. Mol. Ecol. 28, 528–543. doi: 10.1111/mec.14919, PMID: 30375061

