# Supplementary material for: Community Assembly and Co-occurrence Patterns Underlying the Core and Satellite Bacterial Sub-communities in the Tibetan Lakes
Source: Front Microbiol. 2021 Sep 17;12:695465. doi: 10.3389/fmicb.2021.695465 (PMC8567192; doi:10.3389/fmicb.2021.695465)
Supplement: Supplementary file 1 [file Data_Sheet_1.docx]

Supplementary Material

Table S1 The geographic variables data of the 30 Tibetan lakes.

| **Lakes** | **Latitude**  **(N)** | **Longitude**  **(E)** | **Altitude**  **(m)** | **Lake Area**  **(km^2^)** | **Catchment Area**  **(km^2^)** | **Glacier**  **Area (km^2^)** | **NDVI** | **MAAT** |
| --- | --- | --- | --- | --- | --- | --- | --- | --- |
| **Laigu** | 29.3 | 96.82 | 3965 | 8.62 | 2209.9 | 422.33 | 0.32 | 1.97 |
| **Yamdrok** | 28.27 | 90.35 | 4441 | 591.42 | 8125.4 | 70.18 | 0.25 | -8.77 |
| **PMYC** | 28.58 | 90.32 | 5010 | 293.15 | 1766.8 | 122.17 | 0.15 | -1.48 |
| **QYL** | 28.88 | 90.22 | 5130 | 93.00 | 8125.4 | 70.18 | 0.25 | -3.26 |
| **NMC** | 30.5 | 91.05 | 4700 | 2026.74 | 10741.3 | 179.98 | 0.25 | -4.35 |
| **BC** | 31.23 | 91.12 | 4664 | 144.30 | 3321.2 | 22.12 | 0.26 | -1.14 |
| **PE** | 31.43 | 90.92 | 4522 | 176.25 | 3321.2 | 22.12 | 0.26 | -0.15 |
| **ZGTC** | 32.05 | 90.83 | 4561 | 234.10 | 3506.7 | 0.00 | 0.22 | -0.51 |
| **SBC** | 32.02 | 88.25 | 4509 | 87.06 | 3481.9 | 7.83 | 0.14 | 0.31 |
| **DZC** | 31.83 | 87.47 | 4393 | 294.90 | 12848.7 | 42.42 | 0.15 | 0.75 |
| **LC** | 29.21 | 87.38 | 4213 | 8.77 | 93.8 | 0.00 | 0.25 | 1.4 |
| **DJC** | 29.88 | 85.73 | 5130 | 111.31 | 763.6 | 2.54 | 0.15 | -2.83 |
| **GRC** | 30.81 | 84.97 | 4639 | 67.14 | 1053.8 | 0.00 | 0.12 | -3.64 |
| **YBC** | 30.82 | 84.82 | 4643 | 64.90 | 1053.8 | 0.00 | 0.15 | -1 |
| **ZRNMC** | 31.07 | 85.4 | 4595 | 1010.90 | 20080.1 | 71.56 | 0.12 | -0.31 |
| **AGC** | 31.18 | 85.45 | 4658 | 33.30 | 20080.1 | 71.56 | 0.15 | -1.84 |
| **QGC** | 31.2 | 85.48 | 4663 | 20.30 | 20080.1 | 71.56 | 0.15 | -1.05 |
| **QMKC** | 31.23 | 85.07 | 4628 | 9.04 | 2457.7 | 15.35 | 0.16 | -0.71 |
| **DWC** | 31.23 | 85.05 | 4626 | 117.55 | 2457.7 | 15.35 | 0.16 | -0.71 |
| **DC** | 32.12 | 84.73 | 4396 | 14.94 | 525.0 | 0.00 | 0.12 | 0.46 |
| **TRC** | 31.12 | 84.3 | 4566 | 490.32 | 16767.8 | 119.31 | 0.14 | -0.42 |
| **ZBCK** | 31.35 | 84.07 | 4417 | 126.08 | 16767.8 | 119.31 | 0.15 | 0.72 |
| **ALRC** | 31.43 | 83.37 | 4899 | 505.45 | 12463.8 | 135.25 | 0.13 | -1.05 |
| **GZC** | 30.65 | 82.13 | 4710 | 54.35 | 918.4 | 0.21 | 0.14 | -2.7 |
| **RBC** | 33.55 | 79.81 | 4167 | 658.81 | 27524.2 | 590.34 | 0.06 | 0.58 |
| **AIYONG** | 33.37 | 80.55 | 4292 | 21.25 | 1819.1 | 0.00 | 0.11 | 0.66 |
| **BGC** | 33.55 | 79.81 | 4167 | 658.81 | 27524.2 | 590.34 | 0.06 | 0.58 |
| **HL** | 34.47 | 80.42 | 5257 | 12.72 | 401.5 | 0.83 | 0.08 | -6.47 |
| **LONGMU** | 34.58 | 80.37 | 4933 | 103.87 | 957.2 | 9.65 | 0.07 | -7.3 |
| **SUMXI** | 34.58 | 80.23 | 4975 | 29.48 | 1903.9 | 144.82 | 0.06 | -6.85 |

Note: MAAT, mean annual air temperature; NDVI, Normalized Difference Vegetation Index.

Table S2 Bacterial diversity of Tibetan lakes based on 16S rRNA gene.

| **Lakes** | **No. of OTU** | **Shannon-Wiener diversity index H’** | **Chao1** | **Good’s coverage (%)** | **Pielou evenness index J** |
| --- | --- | --- | --- | --- | --- |
| **Laigu** | 261 | 5.15 | 591.55 | 93% | 0.64 |
| **Yamdrok** | 375 | 6.19 | 694.92 | 91% | 0.72 |
| **Yamdrok1** | 238 | 5.40 | 505.96 | 94% | 0.68 |
| **PMYC** | 206 | 5.16 | 463.55 | 95% | 0.67 |
| **PMYC1** | 252 | 5.26 | 679.69 | 93% | 0.66 |
| **PMYC2** | 241 | 5.84 | 376.02 | 95% | 0.74 |
| **PMYC3** | 213 | 5.51 | 365.79 | 96% | 0.71 |
| **QYL** | 320 | 6.13 | 834.72 | 92% | 0.74 |
| **NMC** | 261 | 5.87 | 586.11 | 94% | 0.73 |
| **NMC1** | 265 | 5.71 | 671.12 | 94% | 0.71 |
| **BC** | 205 | 5.54 | 487.55 | 95% | 0.72 |
| **BC1** | 337 | 5.90 | 803.91 | 90% | 0.70 |
| **PE** | 253 | 5.76 | 511.03 | 94% | 0.72 |
| **PE1** | 502 | 7.64 | 713.03 | 90% | 0.85 |
| **PE2** | 318 | 5.99 | 704.82 | 92% | 0.72 |
| **ZGTC** | 266 | 5.58 | 564 | 93% | 0.69 |
| **ZGTC1** | 309 | 5.87 | 739.88 | 91% | 0.71 |
| **ZGTC2** | 607 | 8.24 | 886.35 | 88% | 0.89 |
| **ZGTC3** | 173 | 5.61 | 422.4 | 96% | 0.75 |
| **ZGTC4** | 224 | 5.83 | 643.25 | 94% | 0.75 |
| **SBC** | 206 | 5.68 | 456.14 | 95% | 0.74 |
| **DZC** | 213 | 5.51 | 477.44 | 95% | 0.71 |
| **DZC1** | 261 | 5.52 | 596.52 | 94% | 0.69 |
| **LC** | 152 | 4.26 | 302.47 | 96% | 0.59 |
| **DJC** | 296 | 6.11 | 582.22 | 93% | 0.74 |
| **GRC** | 294 | 5.99 | 526.81 | 93% | 0.73 |
| **YBC** | 299 | 6.19 | 790.13 | 92% | 0.75 |
| **ZRNMC** | 302 | 6.16 | 656.51 | 92% | 0.75 |
| **AGC** | 398 | 7.14 | 469.79 | 94% | 0.83 |
| **AGC1** | 232 | 5.85 | 564.54 | 94% | 0.74 |
| **AGC2** | 324 | 6.33 | 733.64 | 92% | 0.76 |
| **QGC** | 228 | 4.75 | 600.86 | 94% | 0.61 |
| **QMKC** | 179 | 4.81 | 460.89 | 95% | 0.64 |
| **DWC** | 206 | 5.09 | 487.25 | 94% | 0.66 |
| **DC** | 347 | 6.30 | 953.49 | 90% | 0.75 |
| **TRC** | 280 | 6.18 | 615.83 | 93% | 0.76 |
| **ZBCK** | 223 | 4.42 | 423.68 | 94% | 0.57 |
| **ALRC** | 254 | 5.83 | 426.58 | 95% | 0.73 |
| **GZC** | 310 | 6.11 | 548.68 | 93% | 0.74 |
| **GZC1** | 214 | 5.26 | 386.58 | 95% | 0.68 |
| **RBC** | 265 | 6.05 | 683.38 | 93% | 0.75 |
| **AIYONG** | 321 | 6.32 | 924.03 | 91% | 0.76 |
| **BGC** | 205 | 5.54 | 487.55 | 95% | 0.72 |
| **BGC1** | 337 | 5.90 | 803.91 | 90% | 0.70 |
| **HL** | 515 | 7.14 | 1097.71 | 86% | 0.79 |
| **LONGMU** | 179 | 5.02 | 353.84 | 96% | 0.67 |
| **SUMXI** | 348 | 6.46 | 677.91 | 91% | 0.77 |

Table S3 OTU compositions of the core and satellite sub-communities within each phylum.

| Taxonomy |  | Core | |  | Satellite | |
| --- | --- | --- | --- | --- | --- | --- |
|  |  | No. of OTU | Total p_i_ (%) |  | No. of OTU | Total p_i_ (%) |
| *Proteobacteria* |  | 310 | 27.71 |  | 498 | 40.47 |
| *Bacteroidetes* |  | 198 | 21.94 |  | 220 | 17.24 |
| *Actinobacteria* |  | 126 | 21.35 |  | 261 | 20.91 |
| *Cyanobacteria* |  | 26 | 12.83 |  | 55 | 3.69 |
| *Firmicutes* |  | 64 | 4.76 |  | 65 | 4.67 |
| *Tenericutes* |  | 12 | 5.02 |  | 54 | 3.96 |
| *Planctomycetes* |  | 26 | 2.50 |  | 42 | 3.22 |
| *Verrucomicrobia* |  | 18 | 2.17 |  | 21 | 1.4 |
| *Chloroflexi* |  | 9 | 0.58 |  | 14 | 1.04 |
| *Chlorobi* |  | 2 | 0.60 |  | 9 | 0.78 |
| *Gemmatimonadetes* |  | 9 | 0.30 |  | 15 | 1.38 |
| *[Thermi]* |  | 0 | 0 |  | 2 | 0.13 |
| *Acidobacteria* |  | 3 | 0.04 |  | 6 | 0.44 |
| *Candidate_division_TM7* |  | 0 | 0 |  | 2 | 0.11 |
| *Others* |  | 6 | 0.2 |  | 12 | 0.56 |

*pi represents the mean relative abundance.


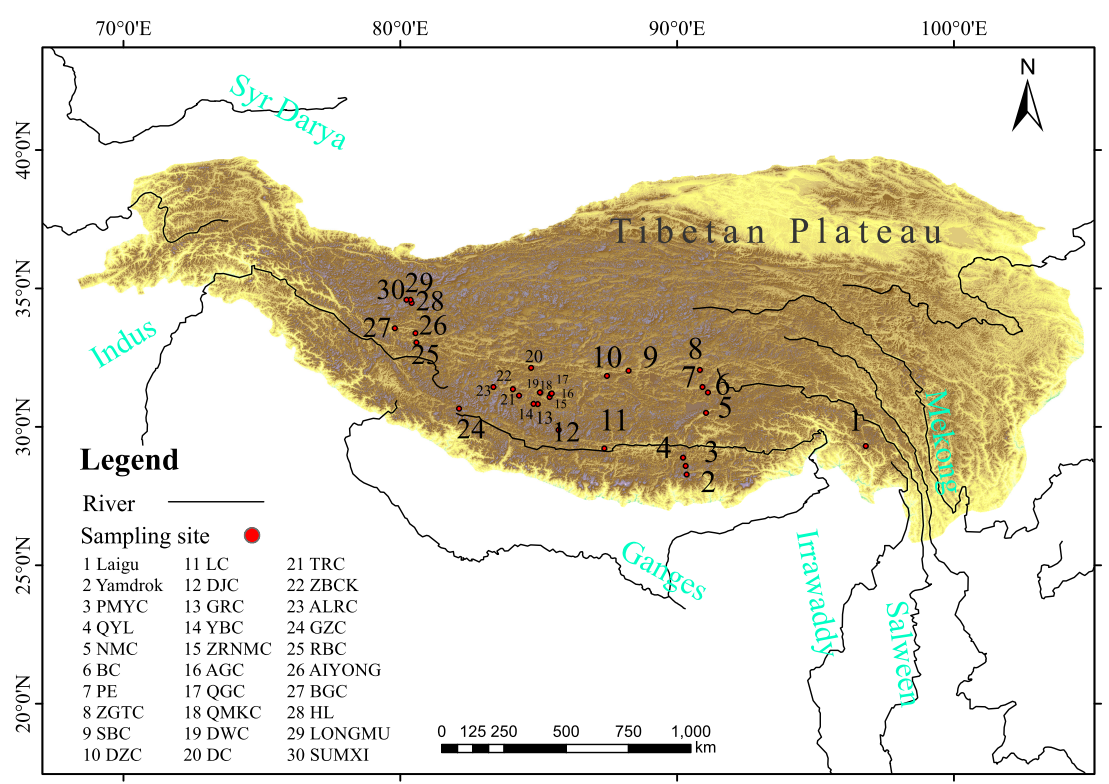
Figure S1 Locations of the 30 sampling lakes located on the Tibetan Plateau.

The code for the lakes is: 1= Laigu Lake (Laigu); 2= Yamdrok (Yamdrok); 3= Puma Yumco (PMYC); 4= Qiangyong Lake (QYL); 5= Namuco (NMC); 6= Bengco (BC); 7= Pengco (PE); 8= Zigetangcuo (ZGTC); 9= Saibucuo (SBC); 10= Dazeco (DZC); 11= Langco (LC); 12= Jiadaco (DJC); 13=Garenco (GRC); 14= Youbuco (YBC); 15= Zharinanmucuo (ZRNMC); 16= Anguco (AGC); 17= Qigeco (QGC); 18= Qingmukeco (QMKC); 19= Dawaco (DWC); 20= Dongco (DC); 21= Taroco (TRC); 22= Zhabuyechaka (ZBCK); 23= Anlarenco (ALRC); 24= Gongzhuco (GZC); 25= RBC (RBC); 26= Aiyongco (AIYONG); 27= Bangongco (BGC); 28= High Lake (HL); 29= Longmuco (LONGMU); 30= Songmuxico (SUMXI)


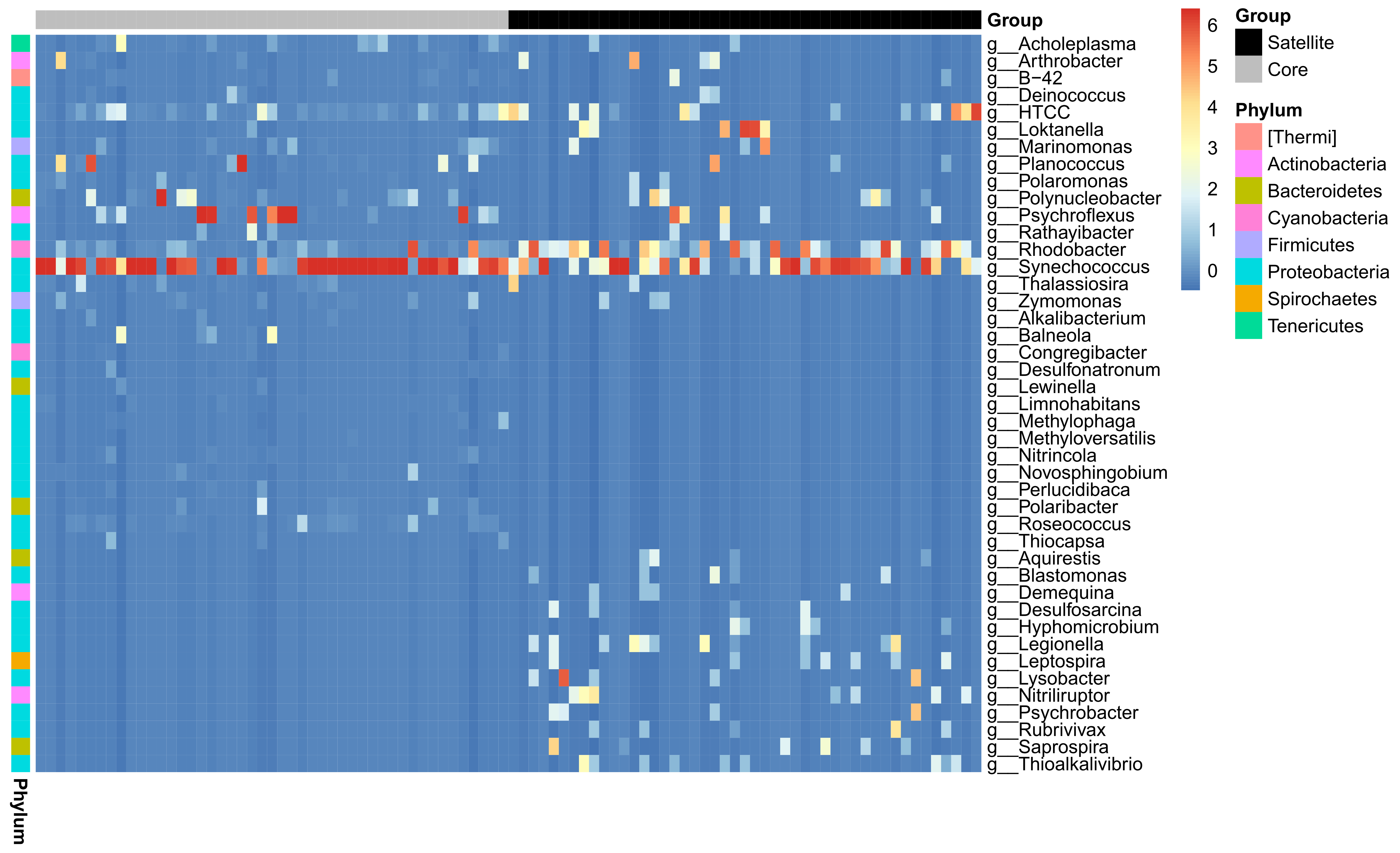


Figure S2 A heatmap showing the distribution pattern of 43 bacterial genera indicates highly significant differences (*P* < 0.05) between core and satellite groups. Each row and column of the heatmap corresponds to a single genus and a sample, respectively. The row data for each genus was transformed by Ln (x + 1). The colors gray and black in the column annotation indicate the core and satellite groups, respectively, and the row annotations on the left side indicate the phylum of each genus.


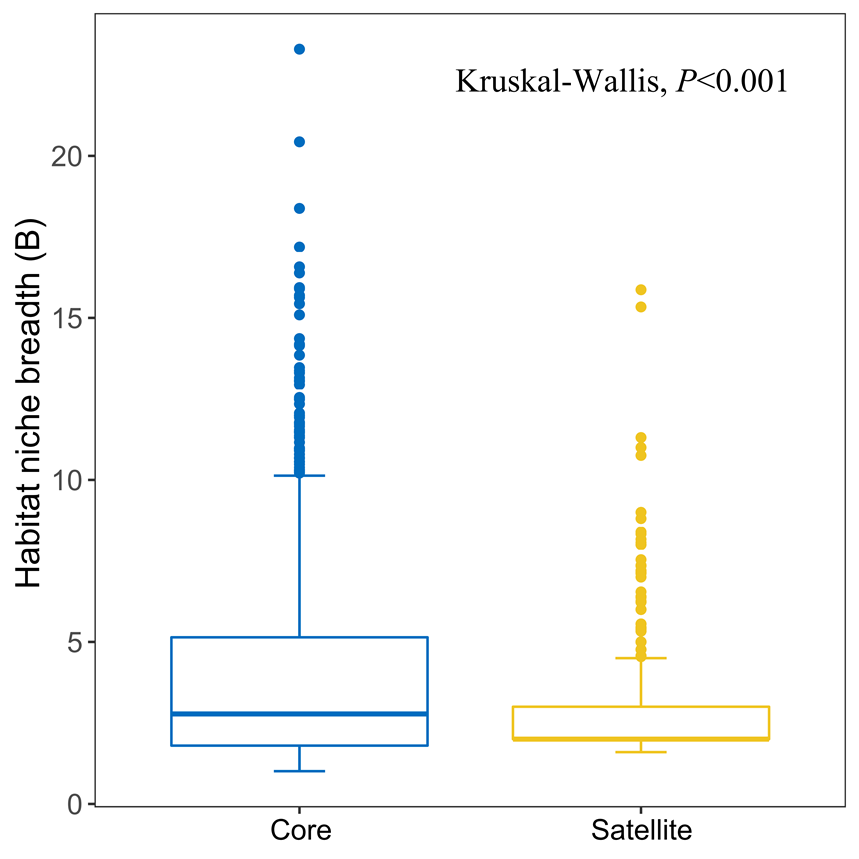


Figure S3 Boxplot summarizing mean niche breadth for the core and satellite sub-communities.


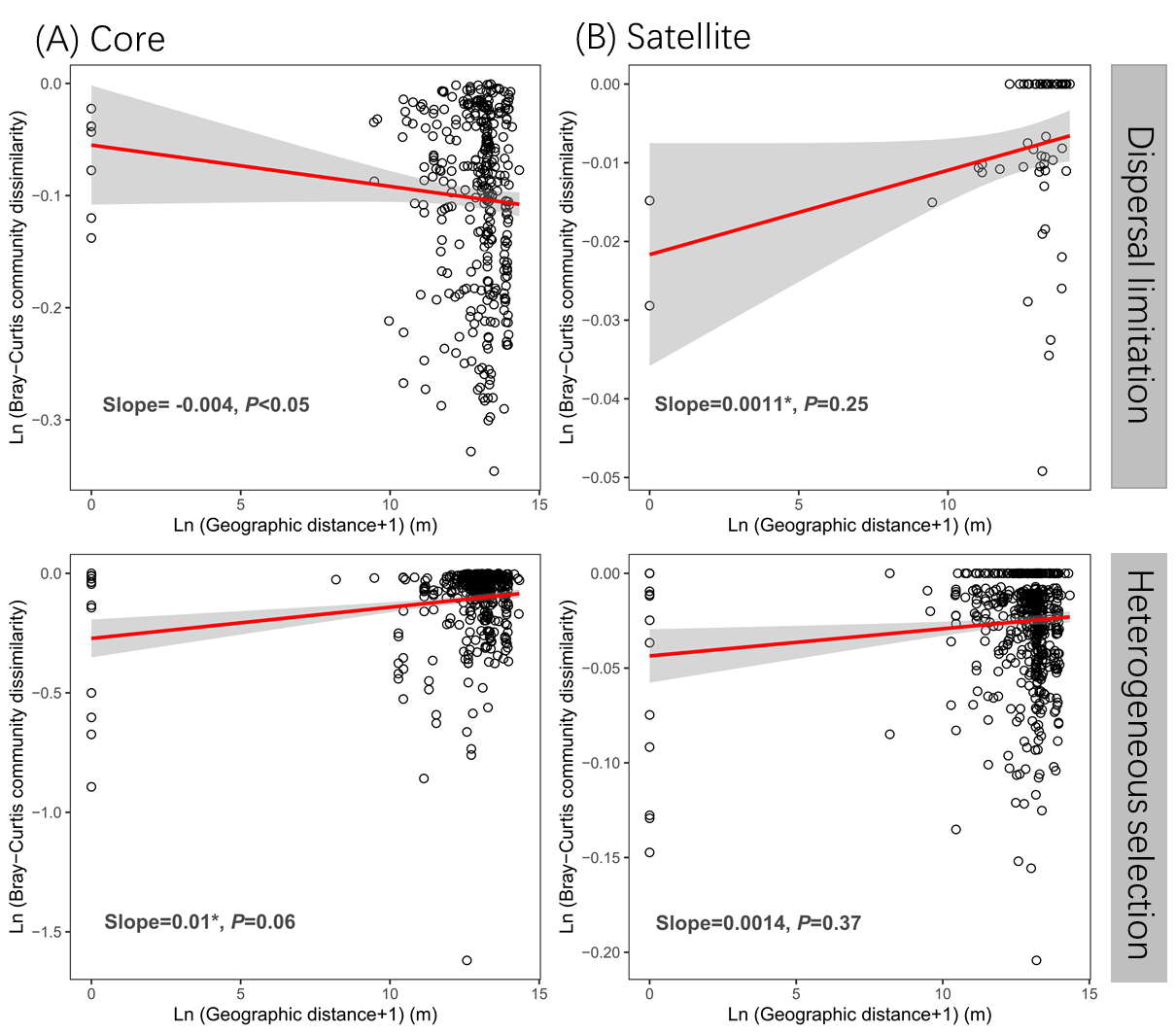


Figure S4 The relationship between geographic distances and Bray-Curtis dissimilarities of the (A) core, and (B) satellite bacterial sub-communities in dispersal limitation and heterogeneous selection processes. The red line in each plot represents a linear regression model fit to Ln (geographic distance+1) versus Ln (Bray-Curtis community dissimilarity). Gray band around the line indicates 95% confidence interval. Asterisks denote significant different between the core and satellite slopes (*P* < 0.05).


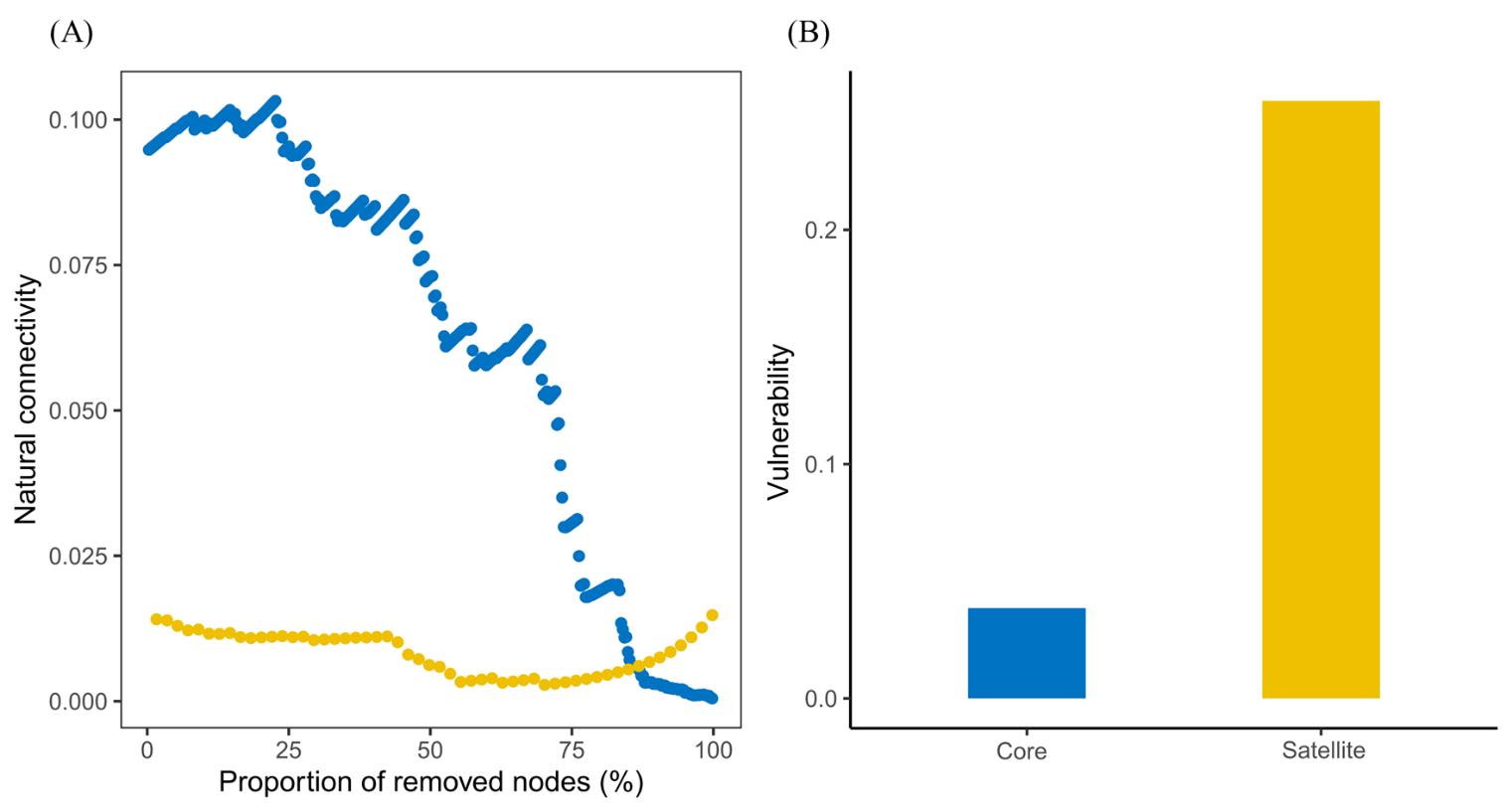


Figure S5 The stability of the core and satellite networks in the Tibetan lakes. (A) Network robustness analysis of the core and satellite bacterial sub-communities. (B) Network vulnerability measured by maximum node vulnerability in the core and satellite network.
